# Patterns of seasonal plasticity in evaporative water loss and preferred temperature in three geckos of the wet–dry tropics

**DOI:** 10.1007/s00442-025-05692-6

**Published:** 2025-03-14

**Authors:** Kimberley Day, Chava L. Weitzman, Angga Rachmansah, Kade Skelton, Keith Christian

**Affiliations:** Research Institute for the Environment and Livelihoods, Charles Darwin University, Ellengowan Drive, Brinkin, NT 0810 Australia

**Keywords:** Acclimatisation, Seasonal plasticity, Evaporative water loss, Thermoregulation, Hydroregulation

## Abstract

Seasonal physiological plasticity (acclimatisation) facilitates homeostasis in changing environments and has been studied extensively with respect to thermal biology and metabolism. Less is known about seasonal changes in evaporative water loss (EWL) in response to changing water availability and humidity. The wet–dry tropics of northern Australia experience moderate seasonal temperature changes, but substantial changes in rainfall and humidity. We studied three gecko species (*Amalosia rhombifer*, *Heteronotia binoei* and *Hemidactylus frenatus*) in the wet and dry seasons with respect to their EWL, preferred body temperatures (*T*_pref_), and their choice between a dry and humid refuge at and below *T*_pref_. EWL was significantly lower in the dry season (66% of wet season values). *T*_pref_ for two of the species did not change seasonally, but *A. rhombifer* selected lower *T*_pref_ during the warmer wet season. Given a choice of refugia, the humid refuge at low temperatures was never preferred over the warm microhabitat. When both refugia were at the preferred temperature, only *A. rhombifer* showed a preference for the humid microhabitat. These results demonstrate that although thermoregulation is prioritised in the short term, hydroregulation (physiological plasticity in EWL) is adjusted in the longer term, with shifts occurring on a seasonal scale. However, it is possible that shifts in EWL may occur in response to prevailing weather conditions on a shorter timescale. Before broad generalisations can be drawn about the phenomenon of EWL plasticity, measurements need to be taken from more species in different climatic regions at ecologically relevant timescales.

**Supplementary Information:**

The online version contains supplementary material available at 10.1007/s00442-025-05692-6.

## Introduction

Physiological plasticity is widespread in nature, maximising performance and survival in response to changing climate and ecological interactions across the year. For instance, plasticity can buffer against the negative effects of increasing temperatures, allowing for increasing heat tolerance in ectotherms to reduce the likelihood of overheating (Gunderson et al. 2017). Though commonly discussed in terms of seasonal temperature changes, flexibility in physiological processes may also be initiated, or optimised, by other environmental cues. Reductions in metabolic rate and preferred body temperature conserve energy and water during seasons when resource availability is limiting even if environmental temperatures are not (Christian et al. 1999a, 2023; Berg et al. 2017). Importantly, the extent or strength of plasticity may depend on the variability experienced by the organism (Muñoz and Bodensteiner 2019). A comparison of 41 bird species from the Central American wet tropics versus six species from a temperate site found a greater seasonal change in the temperate species in thermal and metabolic measures (Pollock et al. 2019). Similarly, variability in resources in the seasonal (wet–dry) tropics may favour the evolution of the physiological plasticity of ectotherms as compared to the relative stability of the aseasonal (wet) tropics (Christian et al. 1999a, 2023; Huey et al. 2012).

Body temperature (*T*_b_) influences all biological processes, including digestion, locomotion, reproduction, and growth and has been widely studied in reptiles and other ectotherms (Heatwole 1976; Huey 1982; Christian and Tracy 1981; Kearney and Predavec 2000; Navas et al. 2008; Chukwuka et al. 2020; Volkoff and Rønnestad 2020 and see below). In seasonal climates, some species become dormant and forego thermoregulation, but other species thermoregulate to lower temperatures in winter (Seebacher 2005). The seasonality of the wet–dry tropics induces an acclimatisation response in some lizards, with thermal preference shifting towards lower *T*_b_ in the cooler dry season compared to the warmer wet season (Christian et al. 1983, 1999b; Christian and Bedford 1995, 1996; Christian and Weavers 1996). However, rather than being a response to environmental temperatures per se, these examples of seasonal changes in preferred body temperature (*T*_pref_) are likely mechanisms to conserve energy and water in response to the decrease in food and water resources in the dry season (Christian et al. 1999a, 2023; Berg et al. 2017). Energy budget analyses indicate that seasonal food availability is the driving force for physiological plasticity related to energy expenditure (*T*_pref_ and metabolic rate) in several lizards from the seasonal tropics (Christian et al. 1996a, 1999a, 1999b), and calculations indicate that frillneck lizards would starve in the dry season were it not for these physiological adaptations (Christian et al. 1996b). Thus, physiological plasticity is likely to be essential for some species in environments with seasonal shortages of food coupled with high environmental temperatures (Christian et al. 2023).

The species described above are diurnal and are thus exposed to more extreme thermal environments than nocturnal species. Nevertheless, geckos can thermoregulate by selecting microhabitats or basking in their daytime refugia (Bustard 1967; Chukwuka et al. 2021; Kearney and Predavec 2000), and night-time habitats maintain some thermal heterogeneity, allowing nocturnal geckos to use warm microclimates to conductively thermoregulate while active (Autumn and DeNardo 1995; Nordberg and Schwarzkopf 2019). Nocturnal insects are substantially less abundant in the dry season compared to the wet season in the seasonal Australian tropics (Churchill 1994). However, the lower body temperatures typically experienced by nocturnal animals from this region (Nordberg and Schwarzkopf 2019) are similar to the daytime *T*_b_s of the diurnal species that have seasonally-reduced *T*_pref_ values (Christian and Bedford 1995; Christian and Weavers 1996; Christian et al. 1996a, 1999b), possibly obviating the need for further decreases in *T*_b_ to conserve energy. Thus, it is difficult to predict whether or not nocturnal geckos would exhibit seasonal acclimatisation of *T*_pref_. Little is known about seasonal thermal acclimatisation in nocturnal species from the wet–dry tropics, but the gecko *Oedura marmorata* selected a lower *T*_pref_ in the dry season compared to the wet (Christian et al. 1998).

While thermoregulation is relatively well-studied, the importance of hydroregulation in reptiles has only recently become better understood (Grimm-Seyfarth et al. 2018; Kearney et al. 2018; Pirtle et al. 2019; Rozen-Rechels et al. 2019). In addition to the potentially lethal consequences of inadequate hydration, sublethal dehydration can result in negative physiological and ecological consequences (Pirtle et al. 2019; Rozen-Rechels et al. 2019). In particular, optimal thermoregulation and activity patterns can be disrupted, resulting in compromised performances related to foraging, predator avoidance, and reproductive success (Rozen-Rechels et al. 2019, 2021; Sannolo and Carretero 2019).

Reptiles from wet or mesic environments typically have higher rates of evaporative water loss (EWL) compared to those from arid environments (Hillman and Gorman 1977; Dmi’el et al. 1997; Cox and Cox 2015), and within a climatic zone, animals occupying mesic microhabitats have higher rates of EWL than those from drier microhabitats (Belasen et al. 2017). The mechanisms behind these patterns are typically not known but include the possibilities of genetic differences among populations, irreversible developmental phenotypic plasticity in response to the environment, or reversible physiological plasticity in which an individual can change in response to environmental conditions (Dmi’el et al. 1997; Wilson and Franklin 2002; Cox and Cox 2015; While et al. 2018; Christian et al. 2023). An example of a reversible change in EWL is related to the morphological and metabolic consequences of pregnancy in a viviparous snake (Lourdais et al. 2017). Pregnant snakes have higher rates of EWL and select warmer and moister microhabitats, further supporting a pattern between EWL and microhabitat humidity, but in this case being driven by biological processes rather than by environmental conditions (Lourdais et al. 2017).

Although EWL occurs across skin, eyes, and respiratory structures, cutaneous water loss is the largest component in reptiles (Bentley and Schmidt-Nielsen 1966; Cohen 1975; Shoemaker and Nagy 1977; Mautz 1982; Kobayashi et al. 1983), typically accounting for more than 70% of the total EWL (Blamires and Christian 1999). Physiological plasticity of EWL has been explored in lizards in the laboratory, with individuals exposed to humid conditions having higher rates of EWL than those acclimated to dry conditions (Kobayashi et al. 1983; Kattan and Lillywhite 1989; Rozen-Rechels et al. 2020; Weaver et al. 2022, 2023). Seasonal changes in EWL have been documented in beetles exposed to semi-natural conditions (Cooper 1985) and, in arid-adapted scorpions exposed to natural conditions, seasonal physiological plasticity in EWL has been mechanistically linked to seasonal changes in epicuticular biochemical composition (Toolson and Hadley 1979). We are only aware of a single published report of field acclimatisation in which lizards measured during a wet season had higher rates of EWL than lizards measured from the same location during a dry time of the year (Blamires and Christian 1999). This study showed no significant seasonal differences in ocular or respiratory EWL, but there was a seasonal change in cutaneous EWL, which is consistent with measurements of skin permeability being higher in lizards acclimated to wet conditions as compared to those acclimated to dry conditions in the laboratory (Kobayashi et al. 1983; Kattan and Lillywhite 1989; Weaver et al. 2023).

While there is growing evidence of seasonal thermal and metabolic plasticity in reptiles (Christian et al. 2023), the focus has been on diurnal species, despite differing ecological pressures on nocturnal versus diurnal habits. Before generalisations can be drawn about the pervasiveness and ecological drivers of acclimatisation, more information is needed from a range of climatic zones (Christian et al. 2023), and this is particularly true for nocturnal animals. Nocturnal geckos are informative in this regard because they can be compared to the better-studied lizard species from the same area, thus providing insight into both the prevalence of physiological plasticity and the ecological drivers. This study aims to quantify seasonal plasticity in the thermal and hydric physiology of three common nocturnal gecko species at a site in the wet–dry tropics in Northern Australia. We focus the study on measuring acclimatisation in EWL and *T*_pref_ in the wet and dry seasons. Given that water availability fluctuates more than the temperature in the wet–dry tropics, we also test for the relative importance of behavioural hydroregulation versus thermoregulation, or the trade-offs between maintaining a preferred temperature or avoiding dehydrating microhabitats. We used this series of physiological and behavioural experiments to address three hypotheses, and although we compared across the species, our over-arching hypothesis was that the climatic conditions of the site was the driving force, and thus the three species would not differ. First, we hypothesised that geckos would decrease EWL to conserve water in the dry season (as per Blamires and Christian 1999). Second, we hypothesised that *T*_pref_ would be lower in the dry season than in the wet as per other lizards in the seasonal tropics (Christian et al. 1983, 1998, 1999a, 1999b, 2003; Christian and Bedford 1995, 1996; Christian and Weavers 1996). Third, we hypothesised that, when provided options of a dry refuge at the preferred temperature and a humid refuge at or below the preferred temperature in the thermo-hydroregulation experiment, prioritisation of desiccation avoidance would emerge at increasing temperatures. That is, geckos would prioritise seeking preferred temperatures when the humid option was at low temperatures, but when offered a humid refuge at higher temperatures, the geckos would shift from a preference for warmth toward a preference for desiccation avoidance (as per Pintor et al. 2016). Thus, under this hypothesis, when both dry and humid refuge options are at the preferred temperature, geckos would show a preference for the humid refuge, which allows both desiccation avoidance and thermoregulation.

## Methods

### Study species, collection site and husbandry

We studied three widespread, common nocturnal gecko species in the wet–dry tropics of Australia: zig-zag geckos (*Amalosia rhombifer*), Bynoe’s geckos (*Heteronotia binoei*) and Asian house geckos (*Hemidactylus frenatus*), as described in Table 1. *Amalosia rhombifer* and *H. binoei* are endemic to Australia, occupying arboreal and predominantly terrestrial habitats, respectively. *Hemidactylus frenatus* is a well-established invasive species from South Asia that became established in Darwin around 1960 (Hoskin 2011) and occupies arboreal habitats. The physiological ecology of *A. rhombifer* has not been studied previously. The *T*_pref_ of *H. binoei* was 30.8°C as measured by individuals from the arid zone of South Australia (Kearney and Porter 2004). In *H. frenatus*, the thermal tolerance to high temperatures does not vary between Thailand and eastern Australia, although thermal tolerance to low temperatures is lower in cooler locations (Lapwong et al. 2021).

**Table 1:** Sample sizes and mass (± standard deviation) of three gecko species included in each physiological experiment

Species		Evaporative water loss		Preferred temperature		Thermo-hydroregulation experiment
		Dry	Wet	Dry	Wet	
*Amalosia rhombifer*	*N*	9 (3F/6M)	16 (9F/7M)	9 (3F/6M)	16 (9F/7M)	9 (5F/4M)
	Mass	1.52 ± 0.212	1.50 ± 0.261	1.52 ± 0.202	1.50 ± 0.254	1.57 ± 0.530
*Heteronotia binoei*	*N*	11 (4F/7M)	6 (6F)	10 (7F/3M)	6 (6F)	7 (2F/5M)
	Mass	2.23 ± 0.568	1.96 ± 0.482	2.33 ± 0.822	1.96 ± 0.446	1.89 ± 0.581
*Hemidactylus frenatus*	*N*	9 (9M)	10 (4F/6M)	10 (2F/8M)	9 (9M)	9 (9M)
	Mass	3.46 ± 0.948	3.53 ± 0.929	3.43 ± 0.942	4.67 ± 0.633	4.53 ± 0.778

Values in parentheses indicate sample sizes per sex

We collected the adult native geckos in the early evening (19:00–22:00) from bushland and *H. frenatus* from both bushland and building walls at Charles Darwin University (CDU), Casuarina, Northern Territory, Australia (12° 22′ 07″ S, 130° 51′ 58″ E). The bushland consists of 5 ha of eucalypt-dominated savanna, adjacent to campus buildings, suburban housing and the Casuarina Coastal Reserve. Sampling was conducted during Darwin’s wet (November–April, with our experiments done from early January–early March) and dry (May–October, with our experiments done from early August–early September) seasons to align with climatic extremes of the wet–dry tropics. In Darwin, > 90% of the annual rainfall (mean = 1722.5 mm) occurs in the wet season, and mean monthly rainfall peaks in January at 429.8 mm and is lowest in July at 1.1 mm. There is some variability of humidity during the short transition periods between the wet and dry seasons, but for most of the wet season, humidity is consistently high, and during the dry season it is consistently low (Online Resource 1; climate statistics from Darwin Airport, 4.5 km south of the study site; www.bom.gov.au/climate/ Accessed 3 October 2024). Mean minimum air temperature decreases by ~ 3 °C in the dry versus wet season, with less variability in maximum air temperatures (Online Resource 1). Geckos were returned to the wild after being run through the experiment(s) within a season, with new individuals captured each season.

Measurements of EWL and *T*_pref_ were taken within 48 h of capture. After that, individuals were fed every second day and provided water via a spray bottle daily. Many geckos in this study were used for two experiments; specifically, all *A. rhombifer* and wet season *H. binoei* in the EWL experiment were subsequently used in the thermal preference experiment, and wet season *H. frenatus* used the thermal preference experiment were also subjected to the thermo-hydroregulation experiment. Following the thermal experiment, lizards were housed in plastic terraria (38 × 23 × 12 cm) with a small hide and placed in a temperature-controlled room set to 28 °C (mean 27.9 ± SD 0.11°C) with a relative humidity of 40.0 ± 1.5% (vapor pressure deficit = 2.2 kPA), with an automated 12 h light–dark cycle until they were used in the thermo-hydroregulation experiment. All measurements were taken within one month, then the animals were released at the site of capture.

### Evaporative water loss

Gecko EWL was measured during the day in the wet and dry seasons for each species using a flow-through system (Blamires and Christian 1999; Young et al. 2005). Evaporative water loss components were plumbed in-line and housed in incubators (model MIR253, Sanyo and model XHC-25, IVYX Scientific) to maintain a nominal experimental temperature of 30 °C (the exact air temperatures were measured by the probes). Five EWL lines were operated simultaneously. For each EWL line, air was drawn from the incubator through a silica gel drying column using a low flow sampler calibrated to 0.2 L min^−1^ (model LFS-113, Gilian^®^). The dry air then passed through an experimental chamber housing the gecko, made from a modified 60 mL syringe (13.5 × 2.6 cm). A probe (model HMP 110, Vaisala™), housed in a plastic tube (30 mm diameter), recorded the relative humidity and air temperature downstream of the gecko. The output from the probes was recorded continuously on an Apple Macintosh computer using an ADInstruments PowerLab paired with LabChart software (model PL3508, ADInstruments Pty Ltd, Bella Vista, Australia), and EWL was calculated from the equations of Bernstein et al. (1977) for an open-flow system, in conjunction with calculations (List 1971) of saturation vapour density (needed to calculate the mass of water from the measurements of relative humidity). In brief, the mass flow of water from the animal:

$$M_{{\text{w}}} = \, V_{{\text{e}}} ({\text{VD}}_{{\text{a}}} {-}{\text{ VD}}_{{\text{i}}} ),$$

where *V*_e_ = experimental flow rate; VD_a_ = water vapour density of the air in the experimental chamber with the animal (g cm^−3^); and VD_i_ = baseline water vapour density (g cm^−3^).

Relative humidity and temperature measurements were collected before and after (as baseline data) measurements with the gecko in the experimental chamber. Skin temperature was recorded from the dorsum of each gecko immediately after the experiment using an infrared thermometer (Traceable© mini, Thomas Scientific, resolution = 0.1 °C, accuracy ± 1 °C). Gecko EWL experiments generally lasted 30 min to 1 h to achieve a resting measure. A flat-line trace on the computer screen was indicative of a resting animal because movement resulted in increased water loss and an irregular trace. The lowest humidity reading over a 2 min period was taken during a rest period of at least 5 min duration. Generally, the readings were very stable while the animals were at rest. If an animal defecated or failed to rest during the experiment, it was re-run later that day. Baseline values were reconfirmed after each experiment.

Total resistance (*R*) to water loss was calculated as: *R* = (VD_s_ − VD_a_) *E*_c_^−1^, where *R* = total resistance to water loss (s cm^−1^), VD_s_ = the vapour density of the skin (taken as the saturation vapour density at the skin temperature, g cm^−3^), VD_a_ = water vapour density of the air in the experimental chamber with the animal (g cm^−3^), and *E*_c_ = the surface area-specific rate of water loss (g cm^−2^ s^−1^) (Spotila and Berman 1976).

Evaporative water loss is a direct function of the surface area over which the flux occurs, and a size-independent measure of total water loss among reptiles of different sizes can be approximated by expressing the total EWL per unit surface area (SA) of the whole animal (Mautz 1982). To calculate gecko SA, we used linear measurements of each gecko (Belasen et al. 2017; Chukwuka et al. 2020). First, the SA of three life-like plastic toy lizard models was determined by covering each model in masking tape, colouring the outer tape with a marker, then carefully transferring the coloured tape to graph paper to obtain a direct measure of SA (Blamires and Christian 1999). Linear measurements were then taken from the same model and substituted into a range of geometric SA equations to determine which one most closely matched the direct measures of SA. Previously, Belasen et al. (2017) and Chukwuka et al. (2020) estimated that lizard SA was roughly equivalent to the body representing a cylinder and the tail a cone. Our estimates with lizard models found that the best geometric SA equation (as compared to the direct measure from the coloured tape, based on the lowest average difference between estimate and direct SA) assumed that the torso (including the head, snout to vent), tail and legs were separate, single-ended cylinders. The length and greatest width of these components were used in calculating SA for each gecko by adding the SAs of the six single-ended cylinders corresponding to the six gecko body parts (Online Resource 2). Body measurements were collected after geckos completed the EWL trial. Alongside linear measurements, geckos were weighed to allow for a seasonal comparison of body condition (see “Statistics” below).

### Thermal preference

Seasonal thermal preference was measured by placing geckos in a thermal gradient with substrate temperatures that ranged non-linearly from 20 to 40 °C and recording *T*_b_ with a thermal camera (model 868, Testo SE & Co. KGaA, Titisee-Neustadt, Germany; Barroso et al. 2016; Nordberg and Schwarzkopf 2019; Sannolo and Carretero 2019). The thermal gradient consisted of an artificial crevice hide made from glazed porcelain tile (54 × 15 × 0.8 cm), supported 1.5 cm above the substrate by a terracotta spacer at each end, and a 50 W heat lamp at one end of the hide, suspended ~ 1 cm above the tile. Each thermal gradient was constructed in a glass tank (modified aquarium, 59 × 34 × 37 cm) with terracotta tiles (56 × 30 × 1.5 cm) as a substrate. Thermal gradients were assembled in controlled temperature rooms set to an air temperature of 19.5 °C, with ten thermal gradients operated simultaneously.

Individual geckos spent a total of approx. 48 h in the preferred temperature experiment, allowing an overnight period to explore the thermal gradient. Thus, the animals had not fed for at least 12 h before the experiment began. Thermal images were collected over the following day and a half at hourly intervals during daylight hours, with a total of 12 thermal images collected from each gecko. Thermal images were processed manually using IR Soft thermal image analysis software (Testo SE & Co. KGaA, Titisee-Neustadt, Germany), with *T*_b_ represented by a measurement from the gecko's dorsum (Nordberg and Schwarzkopf 2019) for each thermal image. For analysis, *T*_pref_ data were reduced to a set point range defined by the central 50% of *T*_b_ measures for each gecko, which represents the target body temperature that an ectotherm tries to achieve (Hertz et al. 1993).

### Thermo-hydroregulation

Geckos were given access to high and low-humidity refugia inside a glass tank (59 × 34 × 37 cm) to determine preference for hydroregulation or thermoregulation across preferred and sub-optimal humidity and temperature combinations (Pintor et al. 2016). Details of the experimental set-up, including a schematic illustration, are given in Skelton et al. (2025). Briefly, they were given a choice between a warm (but dry) refuge and a cool (and humid) refuge, and, in one treatment, a choice between two warm refugia, with one being dry and the other humid. The temperature in the dry refuge remained constant at 32 °C with a mean of 36.5% humidity (absolute humidity (AH) = 12.35 g m^−3^; vapour pressure (VP) = 1.74 kPA; vapour pressure deficit (VPD) = 3.02 kPa) similar to values experienced in the late afternoon in the dry season in Darwin, NT), offering preferred temperature (based on the results of the thermal preference experiment, below) and relatively low humidity. While the dry refuge remained at a constant temperature, the ambient tank conditions and the humid refuge were adjusted to three temperature treatments (32, 27 and 22°C) and a high humidity of 99% in the humid refuge. The 32 °C treatment is close to the *T*_pref_ (see *Results*), and the two lower temperatures are ecologically relevant nighttime temperatures (Online Resource 1). (At 32°C: AH = 33.49 g m^−3^, VP = 4.71 kPA, VPD = 0.05 kPa); at 27°C: AH = 25.52 g m^−3^, VP = 3.53 kPA, VPD = 0.04 kPa; at 22°C: AH = 19.24 g m^−3^, VP = 2.62 kPA, VPD = 0.03 kPa). We refer to these treatments as 32H/32D, 27H/32D, and 22H/32D throughout, indicating the humid and dry (constant) refuge temperatures. Temperatures in the humid refuge were varied between treatments by adjusting the room temperature, while the dry refuge temperature was maintained at 32 °C independently using a heat mat. Thus, when geckos were not in either refuge, they would have experienced the treatment air temperature (22, 27 or 32 °C) and an intermediate humidity, depending on their proximity to the humid and dry refugia.

This experiment used the same setup from the thermal gradient, substituting the hide with two terracotta refugia (15 × 15 × 1.5 cm) at opposite ends of the tank acting as humid and dry refugia. High humidity was achieved by soaking the terracotta refuge in water to humidify the refuge. The second refuge was kept dry by drawing air from a drying column and pumping it into the refuge through aquarium tubing (4 mm) at 0.2 L min^−1^. Refuge conditions were monitored using hygro-thermometer (model 800027, Sper Scientific) probes placed under each refuge, with an electronic display at the back of the tank. Each treatment trial lasted 20 h, with an additional 4 h prior to data collection to allow conditions to stabilise. Six geckos were trialled in separate tanks simultaneously, with treatments consecutively applied in the reverse order for each new set of geckos.

Refuge selection was recorded using a webcam (Logitech) placed in front of the tank, positioned so that the gecko could be seen when occupying either refuge. An Apple MacBook running Evocam software (version 3.6.5, Evological©) was used to create a time-lapse recording refuge selection (interval 10 s, playback 10 frames s^−1^, 320 × 240 pixels) (Evosec GmbH & Co. KG, Germany). Time-lapse recordings were processed using QuickTime Player video playback software to determine the time spent under each refuge for each temperature treatment.

### Statistics

All analyses were run with R v4.3.1 in RStudio v2023.06.2 (RStudio Team 2023; R Core Team 2023). Where relevant, we used Wald’s tests to determine the significance of the car package (Fox and Weisberg 2019) and pairwise posthoc contrasts in emmeans (Lenth 2023). Predictor variables for primary analyses of data from EWL and *T*_pref_ experiments were species, season, sex, species × season, and sex × species unless otherwise stated. Posthoc tests of these analyses allow us to determine how species differ within each season and whether values differ between seasons within a species.

We analysed EWL rates with a general linear model (GLM) with a log-link, with surface area as a covariate. Total resistance to EWL was also analysed with a GLM with a log-link, including gecko mass as a covariate. In addition to exploring seasonal EWL and seasonal *T*_pref_, we further used geckos collected for the EWL experiment to determine if the geckos differed in body condition between the two seasons. As a measure of body condition, we calculated the ratio of mass divided by SVL (Sion et al. 2021). To analyse body condition, we removed gravid females and analysed with an ANOVA using arcsine square-root transformed data to account for the non-normality of ratios. As six *A. rhombifer* (2 in the dry season, 4 in the wet season) were gravid, we separately analysed whether *A. rhombifer* females had EWL rates influenced by the interaction between season and gravid state with a log-link GLM including surface area as a covariate. Only two *H. binoei* were gravid at the time of sampling, which was too small a sample size to analyse.

Preferred temperatures were analysed with a linear mixed effects model with the lme4 package (Bates et al. 2015). Gecko ID was included as a random factor because each individual had six readings included in the data (the central 50% of the 12 *T*_b_ measures). As six *A. rhombifer* (2 in the dry season, 4 in the wet season) were gravid, we separately analysed whether *A. rhombifer* females had thermal preferences influenced by the interaction between season and gravid state.

To assess thermo-hydroregulation for each species, we used binomial generalised linear mixed effects models (GLMM) in the lme4 package (Bates et al. 2015) to test if the proportion of time spent in any refuge (as opposed to the open), and the proportion of refuge time spent in the humid refuge (as opposed to the dry), varied by treatment. Analyses were weighted by total time in the experiment, and total time spent in refugia (for the humid refuge analysis) and included gecko ID as a random variable. Geckos run in this experiment were mostly males (Table 1); the few female *H. binoei* used the refuges within the range of the males, and we performed no analyses based on sex for *H. binoei* or *H. frenatus* (all male). For *A. rhombifer*, analyses of refuge and humid refuge use included sex and the interaction between treatment and sex. Only one female *A. rhombifer* was gravid during this experiment, which was performed within the range of other females of the species. In addition to our analyses of refuge and humid refuge use within species, we further analysed for differences between species, comparing the three species’ refuge use in the 32H/32D treatment with binomial GLMMs, with weights and random factor as above. Lastly, within each treatment for each species, we used tests of equal proportions to detect if time spent in the humid refuge indicated a preference for or against that option. In these tests, we compared values against 0.5 (50:50 per option, i.e., no preference), results of which would suggest trade-offs and prioritisation of water balance and temperature. Within a species and treatment, values significantly below 0.5 indicate a preference for the dry refuge, while values significantly above 0.5 suggest a humid refuge preference.

## Results

### Seasonal water loss

**Fig. 1:**
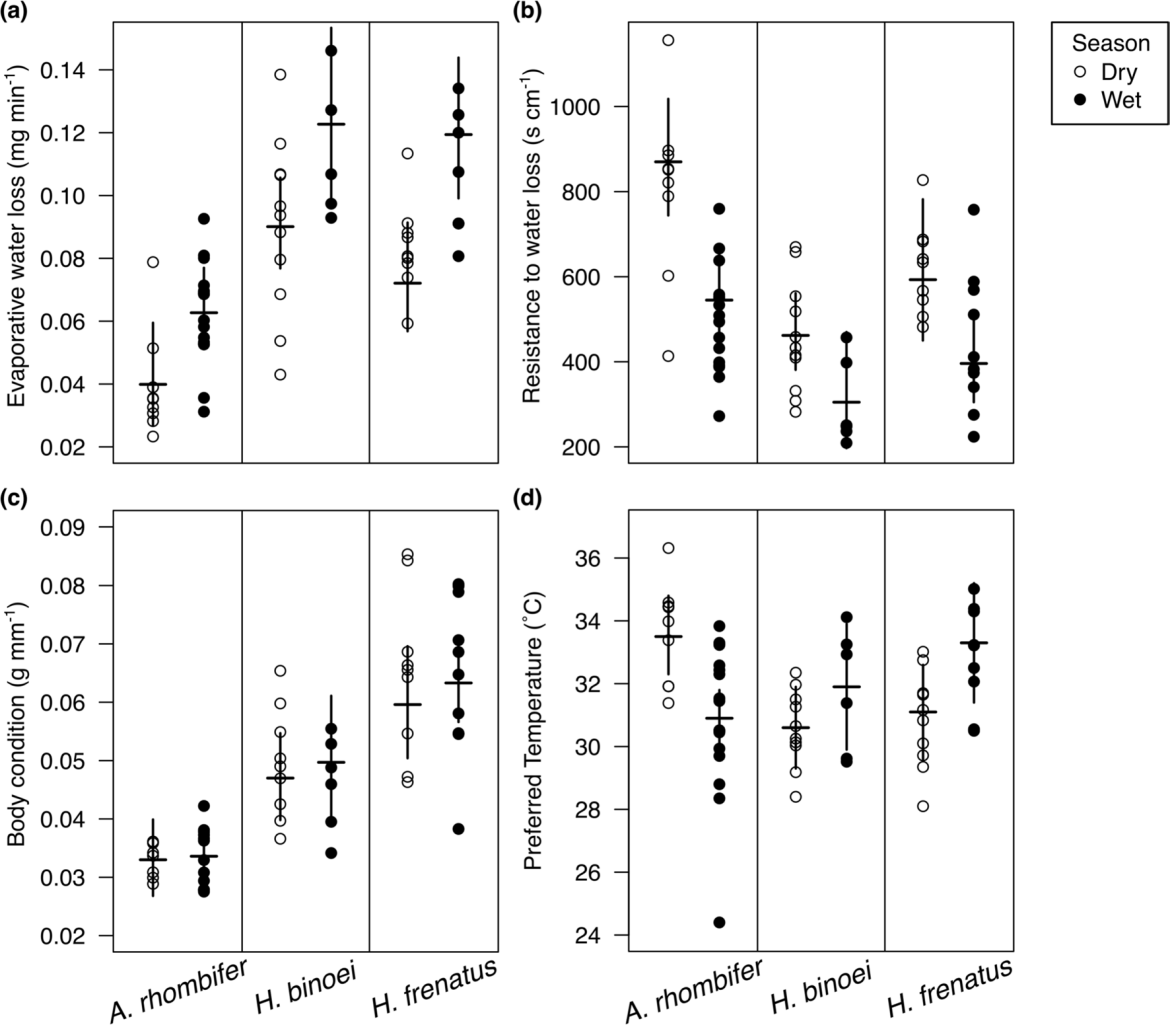
Seasonal differences in EWL and preferred temperatures support acclimatisation in geckos, with no seasonal change in body condition. **a** EWL; **b** resistance to water loss; **c** body condition; and **d** preferred body temperatures. Points are raw values for individual geckos (one average value per gecko for preferred temperature) in the dry (open) and wet (filled) seasons. Horizontal lines denote emmeans estimates with 95% confidence intervals

EWL differed between both seasons and species (Fig. 1a, Table 2), but not their interaction, nor was there a difference between males and females. Dry season EWL rates were lower than wet season rates (65.5% of wet season rates, on average). *Amalosia rhombifer* had lower EWL than the other two species (48–54% of the others; *p* ≤ 0.001 each).

**Table 2:** Results of GLMs (evaporative water loss, resistance to water loss), ANOVAs (T_sdiff_, body condition), and LMMs (preferred temperature) assessing physiological metrics in three gecko species

Response	Predictor	Stat	*df*	*p*
EWL	Surface area	0.017	1	0.9
	Season	5.14	1	**0.02**
	Species	10.24	2	**0.006**
	Sex	0.02	1	0.9
	Season × species	1.03	2	0.6
	Sex × species	2.65	2	0.3
Total resistance to water loss	Mass	3.13	1	0.08
	Season	24.96	1	**< 0.0001**
	Species	11.71	2	**0.003**
	Sex	0.97	1	0.3
	Season × species	0.14	2	0.9
	Sex × species	1.64	2	0.4
*T*_sdiff_	Season	24.66	1.52	**< 0.0001**
	Species	26.86	2.52	**< 0.0001**
	Sex	1.03	1.52	0.3
	Season × species	0.89	2.52	0.4
	Sex × species	0.97	2.52	0.4
Body condition	Season	0.02	1.44	0.9
	Species	3.52	2.44	**0.04**
	Sex	0.05	1.44	0.8
	Season × species	0.08	2.44	0.9
	Sex × species	0.88	2.44	0.4
Preferred temperature	Season	11.42	1	**0.0007**
	Species	7.28	2	**0.03**
	Sex	0.19	1	0.7
	Season × species	18.90	2	**< 0.0001**
	Sex × species	0.57	2	0.8

Bold denotes significant *p* values

As expected, the results of total resistance to EWL were similar to those for EWL rates above (Table 2). Total resistance also differed between both season and species (Fig. 1b), with *A. rhombifer* experiencing 183% the resistance of *H. binoei* (*p* = 0.0001) and marginally higher resistance than *H. frenatus* (142% the resistance of *H. frenatus*, *p* = 0.06). In contrast, *H. binoei* had 78% the resistance of *H. frenatus* (*p* = 0.3). On average, the total resistance in the dry was 154% that in the wet, identifying the capacity for dramatic physiological changes between the seasons.

Among female *A. rhombifer*, gravid state did not predict EWL rates (gravidity: *Χ*^2^ = 0.78, *df* = 1, *p* = 0.4; season × gravid: *Χ*^2^ = 0.36, *df* = 1, *p* = 0.5). However, the data suggest that gravid females (*A. rhombifer* and *H. binoei*) may experience increased rates of evaporative water loss, though larger sample sizes are required to verify this.

### Thermal preference

*T*_pref_ was predicted by season, species and their interaction (Fig. 1e, Table 2) but not sex. Unexpectedly, preferred temperatures were lower for *A. rhombifer* in the wet season than the dry season (2.7 ± 0.80 ˚C lower; *p* = 0.001), while *H. frenatus* had higher *T*_pref_ in the wet compared to the dry (2.2 ± 0.91 ˚C higher; *p* = 0.02). *Heteronotia binoei* did not change its *T*_pref_ between the seasons (*p* = 0.2). In the dry season, *A. rhombifer* preferred temperatures were greater than those of the other two species (*p* ≤ 0.04 each), while none of the species differed in the wet season (*p* > 0.06 each).

Whether or not female *A. rhombifer* were gravid did not affect thermal preferences (gravidity: *Χ*^2^ = 0.07, *df* = 1, *p* = 0.8; season × gravid: *Χ*^2^ = 0.0002, *df* = 1, *p* = 1).

### Thermo-hydroregulation

**Fig. 2:**
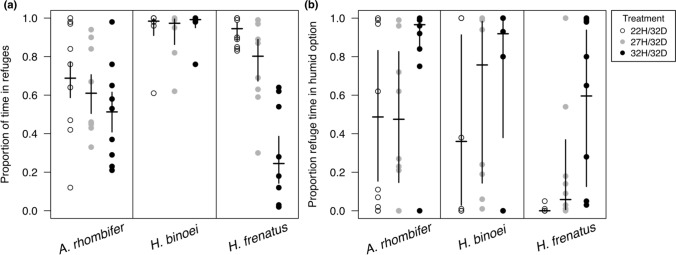
Proportion of time geckos spent in refuges in three temperature treatments. In all three treatments, the dry refuge was 32 °C. Ambient air and the humid refuge were 22, 27, or 32 °C (denoted as 22H/32D, 27H/32D, 32H/32D, respectively). **a** Proportion of total experiment time the geckos spent in either refuge. **b** Proportion of the total refuge time geckos spent in the humid refuge. Points are raw values for individual. Horizontal lines denote emmeans estimates with 95% confidence intervals from binomial GLMMs

*Amalosia rhombifer* had reduced refuge use in increased treatment temperatures (Fig. 2a, Table 3), with less time spent in refugia in the 32H/32D compared with the 22H/32D treatment (51% vs 69% use, respectively; *p* = 0.003). Proportion of refuge time spent in the humid refuge increased at higher treatment temperatures (Fig. 2b), with more time in the humid refuge at 32H/32D compared with the lower two treatments (49%, 48%, and 97% in increasing treatment temperature order; *p* < 0.0001 each). *Amalosia rhombifer* was also the only species to prefer the humid refuge over the dry one, which occurred in the 32H/32D treatment (*p* = 0.046).

**Table 3:** Results of GLMMs of refuge use in three species of geckos in a thermo-hydroregulation experiment

Dependent	Species	Predictor	*Χ*^2^	*df*	*p*
Combined refuge use	*A. rhombifer*	Treatment	11.09	2	**0.004**
		Sex	3.74	1	0.05
		Treatment × sex	1.25	2	0.5
	*H. binoei*	Treatment	4.58	2	0.1
	*H. frenatus*	Treatment	140.2	2	**< 0.0001**
	All, 32 Treatment	Species	26.56	2	**< 0.0001**
Humid refuge use	*A. rhombifer*	Treatment	51.40	2	**< 0.0001**
		Sex	2.44	1	0.1
		Treatment × sex	0.85	2	0.7
	*H. binoei*	Treatment	40.66	2	**< 0.0001**
	*H. frenatus*	Treatment	32.63	2	**< 0.0001**
	All, 32 Treatment	Species	2.07	2	0.4

Bold denotes significant *p* values

Analyses compare the proportion of time geckos spent in either refuge, or the humid refuge, between three temperature treatments and gecko sex. In all three treatments, the dry refuge was 32 °C. For the three treatments, ambient air and the humid refuge were 22, 27, or 32 °C

*Heteronotia binoei* regularly used refugia throughout the experiment (over 90% of the time in each treatment, on average), with no differences among the treatments (Fig. 2a, Table 3). *Heteronotia binoei* did, however, use the humid refuge in differing amounts among the three treatments (*p* < 0.002 each; Fig. 2b), with increasing humid refuge use at increasing treatment temperatures (36%, 76%, 92% refuge time for 22H/32D, 27H/32D, and 32H/32D treatments, respectively). They never exhibited a preference for the humid or dry refuge (*p* > 0.4 each).

In *Hemidactylus frenatus*, refuge use and humid refuge use both differed among the treatments, with decreased overall refuge use in higher treatment temperatures (95%, 80%, 25%, respectively; Fig. 2a), but an increase in proportion of refuge time spent in the humid option (< 1%, 6%, 60% in increasing treatment temperature order; Fig. 2b). All posthoc tests among the treatments were significant (p ≤ 0.0003 each). *Hemidactylus frenatus* only exhibited preference for the warm, dry refuge over the humid refuge in the 22H/32D treatment (*p* = 0.008).

In the 32H/32D treatment, the gecko species differed in their refuge use (Fig. 2a), with *H. binoei* using refuges more than the other species (*p* ≤ 0.0002 each). The species did not differ in the proportion of refuge time that was spent in the humid refuge in this treatment (Fig. 2b).

## Discussion

In this study, we used a series of experiments to assess seasonal acclimatisation and trade-offs between thermal and hydric balances in three gecko species in northern Australia. Supporting our first hypothesis, geckos had reduced water loss, and increased resistance to water loss, in the dry season compared with the wet season. The seasonal changes in EWL in these three nocturnal gecko species are consistent with the acclimatisation response found in a diurnal lizard from the same area in the wet–dry tropics (Blamires and Christian 1999). Although we did not partition EWL into its various components, the large decrease in total EWL during the dry season could only be accomplished by including a substantial decrease in cutaneous EWL. This is consistent with changes in skin permeability observed in laboratory acclimation experiments (Kattan and Lillywhite 1989; Weaver et al. 2022, 2023).

High EWL in the wet season suggests that there is a cost to the maintenance of increased cutaneous resistance (Weaver et al. 2023), which may be the energetic cost associated with lipid synthesis (Kattan and Lillywhite 1989). Although there are obvious advantages to conserving water during the dry season, the environmental factor(s) driving the seasonal change in physiology are not known. Seasonal reductions in the availability of food energy can elicit acclimatisation responses including metabolic depression (Christian et al. 1999a, 2023; Berg et al. 2017) and lower thermal preferences (Christian et al. 1983; Christian and Bedford 1995, 1996; Christian and Weavers 1996)—both of which result in reduced energetic requirements in ectotherms. The limiting resource driving acclimatisation of EWL could be the overall availability of water (including water derived from food and drinking as well as atmospheric water), and therefore hydration state, or it could simply be the availability of water in the air driving changes in skin structure. The fact that body condition did not decline in the dry season (Fig. 1d, Table 2) suggests that sufficient food (and associated water) is ingested during the dry season. Thus, it seems likely that the acclimatisation response resulting in lower EWL during the dry season is in response to low humidity (Weaver et al. 2023).

Our second hypothesis, that *T*_pref_ would be lower in the dry season, was not supported. Although two species exhibited non-significant trends toward decreased preferred temperatures in the dry season, the only significant indicator of acclimatisation suggested inverse acclimatisation in *A. rhombifer* which, contrary to our predictions, preferred warmer temperatures during the dry season. Studies have shown that nocturnal reptiles thermoregulate while active at night and while occupying diurnal retreat sites and will bask opportunistically (Bustard 1967; Kearney and Predavec 2000; Nordberg and Schwarzkopf 2019). Furthermore, careful selection of diurnal retreat sites can enable the exploitation of microclimates and buffer environmental temperatures to maintain preferable *T*_b_ while inactive (Webb and Shine 1998; Chukwuka et al. 2021). Aside from temperature, other environmental factors, such as resource availability, can influence preferred temperature (Smith et al. 2008; Abayarathna and Webb 2021; Christian et al. 2023). The lack of a shift in preferred temperature between seasons, as found in *H. binoei* and *H. frenatus*, has been observed in other reptile species (Hitchcock and McBrayer 2006; Smith et al. 2008; Christian et al. 2023), all of which either live in resource-rich environments with water or are nocturnal.

The seasonal acclimatisation observed in *A. rhombifer* follows an inverse response similar to that identified in a range of physiological traits, including temperature preference, in other reptiles (Autumn and DeNardo 1995; Firth and Belan 1998; Berg et al. 2017). Inverse responses have been attributed to avoidance behaviours, where individuals seek relief from ambient environmental temperatures, evading thermal stress (Firth and Belan 1998). This inverse response may be tied to habitat use by *A. rhombifer*, where frequently perching on branches and shrubs may result in greater exposure to temperature fluctuations. However, sufficient thermal pressure to induce a response is unexpected if suitable retreat sites are available and utilised (Webb and Shine 1998; Chukwuka et al. 2021). Alternatively, a reduction in *T*_pref_ could be a response to lower food availability (Brown and Griffin 2005; Gilbert and Miles 2016). However, the body condition of *A. rhombifer* was not different between seasons, and they have a lower *T*_pref_ in the wet season when insect abundance is high (Churchill 1994). Thus, it seems unlikely that food availability explains the seasonal differences in *T*_pref_ in *A. rhombifer*.

In our experiment involving refugia with different thermal and hydric characteristics, we first examined the use of either refuge (as opposed to being elsewhere in the tank) as a function of temperature. Although the terrestrial *H. binoei* spent most of their time in refugia regardless of temperature treatment, the two arboreal species increased refuge use at low temperatures, as would be expected given that ectotherms are more susceptible to predation at lower body temperatures (Christian and Tracy 1981). Considering periods when one or the other refuge was used, our experiment of preferences between humidity and warm thermal conditions supported our hypothesis, with a shift toward humid refuge use at higher temperatures, although some individuals spent a considerable amount of time in the humid refuge regardless of temperature. The geckos used the humid environment with more frequency as the temperature of that humid refuge increased, such that at suboptimal temperatures, the humid microhabitat was never preferred over the warm one. In fact, at low temperatures, *H. frenatus* preferred the warm, dry habitat, prioritising thermoregulation over hydroregulation. Another Australian lizard, *Carlia rubrigularis*, also prioritised thermal requirements by spending time in dry rather than slightly cooler wet environments, only preferring the wet habitat at temperatures closer to lizards’ preferred temperatures (Pintor et al. 2016). It is likely that short-term water limitations in well-hydrated lizards do not create a state of dehydration critical enough to be immediately addressed by the individual, highlighting the differing time scales that hydric and thermal stressors act on lizards. A study using animals with differing hydration states may have resulted in stronger evidence for hydroregulation.

The interplay between thermoregulation and hydroregulation in natural systems is not well understood, and their associated behaviours may be at odds with each other (e.g., basking in low humidity versus sheltering in high humidity (Pirtle et al. 2019; Rozen-Rechels et al. 2019). Dehydration can influence activity patterns (Davis and DeNardo 2009, 2010) and thermoregulation by reducing basking behaviours and thermoregulation precision, and lead to thermal depression (Ladyman and Bradshaw 2003; Kearney et al. 2018; Rozen-Rechels et al. 2020). However, dehydration in lizards generally occurs over a relatively long time scale (Dupoué et al. 2020), while suboptimal thermal temperatures can have more immediate fitness impacts. High temperatures can result in death in a matter of minutes (Heatwole 1976), and less severe suboptimal temperatures can increase predation risk through reduced locomotor performance, as well as slow digestive rates (Christian and Tracy 1981; Waldschmidt and Tracy 1983). On the other hand, even eight days of water restriction only minimally increased the use of a wet shelter by another small lizard (*Zootoca vivipara*; Chabaud et al. 2023). Although several days of dehydration elevate stress responses, it can also enhance some innate immune functions (Moeller et al. 2017; Brusch et al. 2019) and is unlikely to be lethal in most reptiles (Minnich 1982). Longer exposures to dry conditions, however, resulted in shifts in habitat selection to mitigate dehydration in vipers (Dezetter et al. 2023). Together, these studies demonstrate that thermoregulation and hydroregulation work on different time scales, with thermal requirements having greater importance on a short time scale, and hydric requirements being dealt with on a longer time scale, as evidenced by seasonal acclimatisation of EWL in the geckos of this study.

During prolonged dry periods, some reptiles exhibit decreased body conditions (Davis and DeNardo 2009, 2010). Though water is not readily available during the dry season in the present study’s sampling site, no seasonal change in body condition suggests food availability is sufficient year-round. Although low compared to the wet season (Churchill 1994), measurements of dry season insect abundance have shown that significant numbers of flying insects are active in the first few hours after twilight, which overlaps with nocturnal gecko activity (Bustard 1967; Milne et al. 2005; Lei and Booth 2014). Although we do not have direct measurements of seasonal activity, the three species in this study were active throughout the year without obvious changes in habitat use or behaviour. This contrasts with some diurnal lizards, which show decreased levels of activity during dry periods (Christian et al. 1996a, 1996b, 1999b, 2003; Weaver et al. 2024), with notable exceptions being those that live near water (Christian and Weavers 1996). Mean minimum temperatures in the dry season are also within the thermal foraging range observed for *H. frenatus*, which, alongside the native nocturnal gecko *Gehyra variegata*, is as low as 18 ˚C (Bustard 1967; Lei and Booth 2014). Therefore, temperature is expected to have a negligible impact on foraging in this environment. Thus, the ability to thermoregulate, moderate environmental temperatures, and sufficient food availability throughout the year may lessen the advantages of seasonal physiological plasticity in thermal preference.

The phenomenon of seasonal changes in EWL has implications for the effects of climate change and management decisions related to biodiversity conservation (Seebacher et al. 2015). Thus, it is important that we increase our understanding of the role of habitat variability, the mechanisms, the apparent costs, and the time required for physiological adjustments. The wet–dry tropics, in which humidity and the availability of water change more substantially across seasons than do environmental temperatures, may represent one end of a continuum of environments that favour EWL seasonal plasticity. Wet tropical climates may be at the opposite extreme (Huey et al. 2012; Christian et al. 2023). It is less clear whether or not temperate climates would be conducive to the evolution of EWL plasticity because winter inactivity may obviate the need for seasonal shifts in skin permeability. However, the discovery of an acclimation response after only 8 days (Weaver et al. 2022, 2023) raises the possibility of short-term adjustments related to prevailing weather conditions as opposed to the months-long seasonal pattern we found in the seasonal tropics. Seasonal measurements, or even more frequent measurements, from additional species in a range of environments are required to answer these questions to provide a comprehensive understanding of plasticity in EWL.

## Supplementary Information

Below is the link to the electronic supplementary material.

Supplementary file1 (XLSX 15 KB)

Supplementary file2 (PDF 70 KB)

Supplementary file3 (XLSX 45 KB)

## Data Availability

The raw data for this manuscript are in Online Resource 3.
