# Supplementary material for: Patterns of seasonal plasticity in evaporative water loss and preferred temperature in three geckos of the wet–dry tropics
Source: Oecologia. 2025 Mar 14;207(3):53. doi: 10.1007/s00442-025-05692-6 (PMC11909027; doi:10.1007/s00442-025-05692-6)
Supplement: Supplementary file 2 — Supplementary file2 (PDF 70 KB) [file 442_2025_5692_MOESM2_ESM.pdf]

Patterns of seasonal plasticity in evaporative water loss and preferred temperature in three geckos of the wet–dry tropics

Kimberley Day, Chava L. Weitzman, Kade Skelton, Angga Rachmansah, Keith Christian*

Charles Darwin University, Research Institute for the Environment and Livelihoods, Ellengowan Drive, Brinkin, NT 0810 Australia

* Corresponding author: Charles Darwin University, Research Institute for the Environment and Livelihoods, Ellengowan Drive, Brinkin, NT 0810 Australia; Keith.Christian@cdu.edu.au

Surface area calculations:

To calculate gecko surface area (SA), we used linear measurements of each gecko. The following body parts were treated as one-ended cylinders: torso (including the head, snout to vent), tail, 2 front legs and 2 hind legs. The length and greatest width of these components were used in calculating SA for each gecko by adding the SAs of the six single-ended cylinders corresponding to the six gecko body parts.

Equation for a one-ended cylinder:

$$SA = 2\,\pi\,(0.5\ W)\ L + \pi\,(0.5W)^2$$

Where:

L = length (mm)

W = width (mm)
